# Serosurveillance of Health Care Workers in a COVID Hospital: Immune Response, and Its Longevity

**DOI:** 10.7759/cureus.14020

**Published:** 2021-03-21

**Authors:** Minakshi Mishra, Rajan Chaudhry, Farah Rana, Deb Sanjay Nag, Sudhir Rai

**Affiliations:** 1 Pathology, Tata Main Hospital, Jamshedpur, IND; 2 Surgery, Tata Main Hospital, Jamshedpur, IND; 3 Anesthesiology, Tata Main Hospital, Jamshedpur, IND

**Keywords:** healthcare workers, seroprevalence, sars-cov-2, immunological response, covid-19, antibody

## Abstract

Objective: We aimed to study the seroprevalence of coronavirus disease 2019 (COVID-19) and sustainability of the immune response in health care workers (HCWs). A cross-sectional study was conducted between October 7 and November 30, 2020, in a multi-specialty hospital in Eastern India designated as COVID hospital during this pandemic. Study participants included 2,110 HCWs, including those who have recovered from COVID infection.

Method: HCWs were required to complete a questionnaire and give written consent to participate in the study. Their venous blood sample was collected for serum analysis of IgG antibodies to severe acute respiratory syndrome coronavirus 2 (SARS-CoV-2) by chemiluminescent immunoassay.

Results: Positive IgG antibodies were seen in 924 participants with a point prevalence of 43.79%. Slightly higher reactivity was seen in males. History of COVID-19 infection was noted in 10.9%, with the highest antibody response in 81% cases. A maximum of 87.9% reactivity was seen in the first two months, and a significant fall was noted in the fourth month, with reactivity seen in only 50% of the study participants.

Conclusion: SARS-CoV-2 infection is associated with a variable immune response in the infected population. The declining trend of the antibodies correlates with short-lived protective immunity and the possibility of re-infection. Further studies are needed to explore the probable reasons for varied seroprevalence.

## Introduction

Coronavirus disease 2019 (COVID-19) caused by severe acute respiratory syndrome coronavirus 2 (SARS-CoV-2) infection has impacted nearly 219 different countries affecting more than 101 million people globally [1]. Studies on the immunological response following the entry of the virus and targeting receptors expressed especially on the respiratory epithelial cells have shown an interplay between the antigen-presenting cells, innate and adaptive immunity with key roles played by the B and T memory cells [2]. CD8 cytotoxic T lymphocytes and natural killer cells are essential for an appropriate antiviral response, and memory CD8 T cells are capable of providing protection against secondary infections. In a majority of subjects, the antibody response is seen within the first three weeks of the disease and has been shown to correlate with the severity of the infection [2-4].

Antibodies usually develop within three weeks after infection and bind to the viral proteins for destruction by other immune cells [2,3]. The innate and T cell adaptive immune response and persistence have been found to be variable, and studies have shown that memory B and T cells can persist for more than six months in patients who have recovered from coronavirus infection, after which the cell count is observed to decline gradually [4]. These neutralizing IgG antibodies are considered vital for short-term protection against the virus and reduce the chances of a second infection [5]. Serological surveillance is important for the public health response to this emerging infectious disease which can be attributed to its high infectivity, the prevalence of presymptomatic and asymptomatic transmission, which adds to an increase in infection-related burden [6]. Several serological surveys have been done from an epidemiological point of view and to assess the extent of asymptomatic transmission [7-10]. Health care professionals working at the front line play an important role in providing care and support to patients infected with coronavirus [7]. Those working in close proximity to patients infected with coronavirus are considered to be comparatively at a higher risk of infection and more likely to have higher seroconversion [7,11]. Studying the prevalence of antibodies among healthcare workers (HCWs) in different areas is crucial to understanding the potential risk of transmission, the prevalence of herd immunity, vaccine deployment and risk stratification in different areas of work [2]. Serosurveillance has been done in many different cohorts with prevalence ranging from 2.67% to 24.4% [8,12]. This article is a cross-sectional study on the serological survey conducted shortly after the peak infection among 2110 HCWs working in different areas of a multi-specialty hospital in Eastern India designated as a COVID hospital during this pandemic. Nearly 6000 new positive cases had been detected in the hospital during the study period between 7th October to 30th November 2020.

## Materials and methods

Study design

Institutional approval was taken to study the prevalence of SARS-CoV-2 antibodies among the HCWs working in different areas of the hospital, delivering care and support to the patients admitted to our hospital with COVID-19.

Study population

The hospital employs about 2500 HCWs of which 2110 (84.4%) were included in our study based on the inclusion and exclusion criteria. The HCWs providing direct medical support and clinical care to the patients comprised doctors (n= 249, 11.8%), nursing staff (n= 469, 22.2%), hospital attendants and ward secretaries (n= 548, 26%). Hospital staff who were not in direct contact with patients with coronavirus infection included laboratory technicians (n= 52, 2.5%), security staff (n= 166, 7.9%), administrative staff (n= 184, 8.72%), kitchen and allied staff (n= 290, 13.7%), physiotherapists, pharmacy and hospital enquiry/registration staff (n= 50, 2.4%). 

Inclusion Criteria

All HCWs working in the hospital, either in direct or indirect contact with the patients infected with coronavirus, were included in the study. The participants were included irrespective of age, gender, presence of co-morbidities, and having a history of being tested positive for coronavirus infection.

Exclusion Criteria

HCWs who were not present in the hospital due to any reason during the study period were excluded from the study. Some HCWs who withdrew their consent to participate in the study were not included in the final determination and analysis of results.

Questionnaire

The questionnaire comprised details about age, gender, profession, symptoms of flu-like infection, underlying chronic medical conditions, including cardiovascular, respiratory, and diabetes mellitus (see Appendices). The forms were filled with personal details, and any history of being tested positive for COVID-19 by reverse transcriptase-polymerase chain reaction (RTPCR) and rapid antigen test (RAT) was noted. The subjects gave written consent to participate in the study. The study was conducted from October 7 to November 30, 2020.

Sample collection and testing protocol for SARS-CoV-2 IgG antibodies in serum

The HCWs were informed about the conduction of the COVID-19 antibody serological test by the human resources team, which had prepared a list of all the hospital employees based on their category, area of work, and other details. The HCWs were given specific time slots for these tests. A well-spaced designated area was arranged for this purpose with three sample collection points. Three support staff were assigned the responsibility to assist with the registration formalities. Three phlebotomists were responsible for sample collection. The samples were collected during the normal working hours of the hospital. Social distancing was maintained at all times, and sanitization procedures were strictly adhered to. After every two hours, the samples were transported to the biochemistry laboratory, maintaining the cold chain with a temperature of 13-15 degrees Celsius before being processed and tested.

From each subject, 3 ml of random venous sample was collected for serum analysis of IgG antibodies to SARS-CoV-2. The samples were centrifuged at 4500 rpm for five minutes to collect the serum separately. The detection of antibodies to SARS‑CoV‑2 was done using chemiluminescent microparticle immunoassay in a fully automated immunoassay analyzer (Chemiluminescent immunoassay-CLIA DXI; Beckman Coulter Inc., Brea, CA). The sensitivity and specificity of these tests have been reported to be 100% and 98.6%, respectively (as mentioned in the literature provided with the testing kit). The assay targets the spike glycoprotein (S), N terminal S1 unit receptor-binding-domain of the coronavirus [13].

The results were reported as reactive when the value obtained was greater than one signal-to-cutoff (S/CO) ratio and equivocal when values obtained were between 0.8 and 1 S/CO. Values lower than 0.8 S/CO units were considered non-reactive.

The data was taken from the IT-supported hospital management system and person information line list (maintained on Microsoft Excel (Microsoft® Corp., Redmond, WA)) with results of RTPCR, RAT, and COVID-19 antibody test throughout the study period.

Statistical analysis

Statistical analyses were done with the Statistical Package for Social Sciences (SPSS) software version 23.0 (IBM SPSS Statistics, Armonk, NY). One-way analysis of variance (ANOVA) was used to compare the levels of antibodies among groups. Paired t-test was used to compare the seroconversion time for IgG antibody results. P-values ≤ 0.05 were interpreted as statistically significant.

## Results

A total of 2110 subjects were enrolled in the study following the completion of the study questionnaire. The test for SARS-CoV-2 IgG antibodies revealed that 924 subjects had IgG antibodies against the virus, implying that the prevalence among the staff was 43.8%. These values have been shown in Table 1.

**Table 1:** Distribution of males and females in different categories of antibody levels.

SARS-CoV-2 IgG levels	Males (% of n)	Females (% of n)	TOTAL (% of n)
<0.8 (Non-reactive)	644 (30.5%)	499 (23.7%)	1143 (54.2%)
≥0.8- <1 (Equivocal)	26 (1.2%)	17 (0.8%)	43 (2.0%)
≥1 (Reactive)	478 (22.7%)	446 (21.1%)	924 (43.8%)
TOTAL	1148 (54.4%)	962 (45.6%)	2110 (n)

Of the recruited HCWs, 10.9% (n = 231) reported a history of being tested positive for COVID-19 either by RTPCR (n= 198, 9.4%) or RAT (n= 33, 1.6%). Only 80.9% (n=187) of these participants showed positive reactivity for SARS-CoV-2 antibody tests, while equivocal results were noted in 3% of the participants (n =7). About 16.02% of the subjects (n=37) showed no seroreactivity at all. These results are shown in Table 2.

**Table 2:** Distribution of HCWs with a history of COVID-19-positive status and fully recovered at the time of sampling with respect to SARS-CoV-2 IgG antibody status.

SARS-CoV-2 IgG level	COVID-19 Positive History			No History of COVID-19 (% of n)	TOTAL (% of n)
	RTPCR proven (% of n)	RAT proven (% of n)	Total (RTPCR+RAT) % of n		
						<0.8	32 (1.5%)	5 (0.2%)	37 (1.8%)	1106 (52.4%)	1143 (54.2%)
(Non- reactive)											
≥0.8- <1	5 (0.2%)	2 (0.1%)	7 (0.3%)	36 (1.7%)	43 (2.0%)
(Equivocal)					
≥1	161 (7.6%)	26 (1.2%)	187 (8.9%)	737 (34.9%)	924 (43.8%)
(Reactive)					
TOTAL	198 (9.4%)	33 (1.6%)	231 (10.9%)	1879 (89.1%)	2110 (100%)

**Figure 1:**
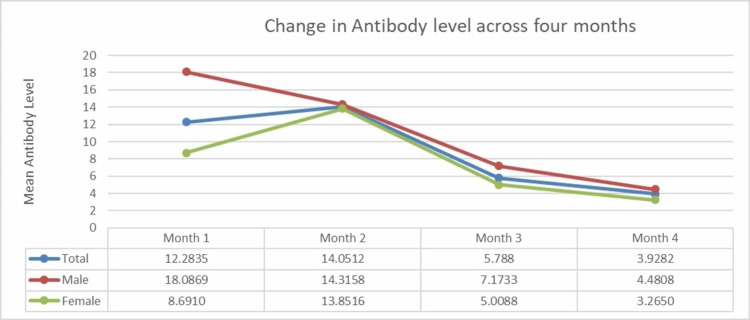
Declining trend of mean antibody level through a period of four months.

The HCWs with reactive antibodies were grouped into categories of four months based on the duration of antibody levels from the date of testing positive for coronavirus infection, confirmed by the results of RTPCR and RAT. Table 3 shows the distribution of the numbers of HCWs month-wise in these categories. A maximum of 87.9% reactivity was seen in the first two months, followed by 78.2% in the third month and a significant fall in the fourth month, with reactivity seen in only 50% of the study participants. A negative correlation (Pearson's coefficient = -0.272) was found between the mean antibody levels and the number of months, which was significant at the 0.01 level (see Appendices). The antibody reactivity was seen the highest in 80.9% of the past coronavirus infection group (10.9% of the total subjects). Males showed a better and sustained immune response as compared to females, as shown in Figure 1.

**Table 3:** One-way ANOVA showing a significant difference between the mean values of the result with a slight increase in antibody from the first month to the second month and a sharp decrease in the third and fourth months.

Months	N	Mean	Std. Deviation	Minimum	Maximum	F-value	p-value
								1	34	12.2835	15.60142	0.02	62.53	8.241	0.0000315
2	100	14.0512	15.13396	0.02	59.96										
3	75	5.788	7.80982	0.03	43.22										
4	22	3.9282	7.23244	0.02	32.57										
Total	231	10.1441	13.23275	0.02	62.53		

## Discussion

SARS-CoV-2 has been associated with significant morbidity and mortality and has adversely impacted global health and economic conditions [6]. Emphasis is placed on the importance of a diagnosis to be made as early as possible [11]. Measures have also been taken to encourage people to improve their immunological status in order to reduce the severity of the infection, protect against other strains of coronavirus and avoid possible re-infection [3]. Studying the kinetics of SARS-CoV-2 antibodies would be helpful in epidemiological surveys, especially in the diagnosis of asymptomatic carriers and to study the impact of COVID-19 infection at the community level and monitor trends in the virus transmission [4].

The present cross-sectional study on HCWs wherein 2110 subjects were tested for the SARS-CoV-2 antibodies showed that 924 subjects developed IgG antibodies with a point prevalence of 43.8% and a slight male preponderance (1.2:1). Coronavirus infection was reported in 10.9% (n =231) persons which had been confirmed with RTPCR and RAT. The highest antibody response was seen in 81% within this group. There was variation in the antibody levels with the duration of coronavirus infection. Using one-way ANOVA a significant difference between the mean values of the result was noted, with a slight increase in antibody levels from the first month to the second month and a sharp decrease in the third and fourth months with a significant p-value.

Variable seroreactivity has been reported in the literature, ranging from 57% prevalence in Bergamo-Italy’s epicenter, 20% in New York City, 5.2% in Kenya, to a low percentage of 4.7% in Los Angeles County and 2.8% in Santa Clara County California [10,14-17]. Overall SARS-CoV-2 seroprevalence of 24.4% in HCWs was significantly greater than the 6% seroprevalence in the general population of the Midlands region, according to data published by Public Health England which suggests a marked occupational risk of exposure for HCWs during the COVID-19 pandemic [9]. Another study found 13.7% IgG prevalence among 40,329 HCWs in the greater New York City area similar to the community prevalence in New York State (14.0%) [15]. Hains et al. [18] reported a seroconversion in 44.0% (n=11/25) of HCWs at a dialysis unit in America. A study in Mumbai on 244 HCWs reported that the prevalence of infection in asymptomatic persons was 4.3% and in previously symptomatic untested HCWs was 70% [19].

Recent research indicates the role of innate and cross-reactive adaptive T cell mediated immunity, which could lower the susceptibility to COVID-19 infection in some individuals and one could speculate the greater frequency of exposure to such agents occurring in HCWs [20]. The other important question is whether HCWs with previous infection or measurable antibodies are immune to reinfections and hence, can be deployed in high‑risk areas [11]. While some initial studies have shown that 40% of asymptomatic subjects and 12.9% of symptomatic subjects became seronegative for IgG antibodies in the “early convalescent phase”, there are other isolated reports of rapid decay of IgG antibodies in persons with mild infection [21-24]. The longevity of the immune response and the level of neutralizing antibodies needed for protection remain unclear, as cases of relapse/reinfection with SARS-CoV-2 have been reported [21].

The variation in the immune response could be attributed to the symptomatology, being less in milder infections, the constitution of individuals, or nutritional status [25]. Further studies are needed to better understand the possible relationship between these factors and variation in the development of antibodies.

The small sample size and absence of serial sampling of the same individual are limitations of the study. Larger serosurveys in HCWs and comparing them to the general population will help in further defining the epidemiology of the illness. At the same time, the presence of antibodies in HCWs should not be equated with immunity or allow for the relaxation of infection control guidelines and practice [24].

## Conclusions

This study was conducted on a one-time basis wherein the HCWs employed at the hospital were tested for the presence of antibodies irrespective of being tested positive for SARS-Cov-2 infection. The declining trend of IgG antibodies with respect to the passage of time in the study participants is indicative of how short-lived the immune response is to coronavirus infection. The findings support the need for active vaccination strategies, prioritizing HCWs who are at a considerably high occupational risk of contracting the infection. This would ensure better protection against the virus by boosting immunity in order to generate a more sustained response. This would not only lower the chances of future infection in the previously uninfected population but also protect against the chances of any possible re-infection. The authors recommend further longitudinal cohort studies with a serial sampling of the same individual to study the longevity of the protective antibodies and potential reasons for varied seroprevalence.

## Appendices

Questionnaire

SERO-SURVEILLANCE HEALTH CARE WORKERS, TMH

JAMSHEDPUR

1) NAME-____________                                                             AGE-      ___                                                        SEX-_______

MR No-_____________

2) Health Profession-

3) Any co-morbid Condition-(please tick)-

                A)DM          B)HTN         C)COPD          D)PREGNANCY       E)CKD            F)CLD(chronic liver disease)

               G) Others (specify)

4) Any Medication-

5) Symptomatic/Asymptomatic-

6) Admitted - YES/NO              Date of admission -----------------------      Date of positivity ------------------------

7) Duration of work in respective work place before turning positive

8) COVID-19 prophylaxis -    HCQ - full dose/partial                                  Ivermectin- full dose/partial

9) Job Profile-

·         Covid sample collection-

·         Suction / intubation-

·         Specimen transport/ handling-

·         Patient care-

·         House keeping-

·         Patient transport-

10) PPE-

·         Compliance 100% -

·         Partially used-

·         Never used-

11) Family History-

·         Any family member with COVID - Yes /No  (If Yes then HCW/Non-HCW)                                    

·         Contact history- 1) patient area                                    2) Colleague

I understand that by signing this document, I am authorizing Tata Main Hospital, Jamshedpur, to carry out antibody testing of my blood for SARS-CoV-2. The test result may be used for sero-surveillance.

THANK-YOU FOR YOUR TIME                                                             Signature

Correlation

**Table 4:** A significant negative correlation was observed between the number of months and mean antibody levels. **. Correlation is significant at the 0.01 level (2-tailed).

		Number of months	Mean antibody levels
Number of months	Pearson Correlation	1	-.272^**^
	Sig. (2-tailed)		.000
	N	210	210
Mean antibody levels	Pearson Correlation	-.272^**^	1
	Sig. (2-tailed)	.000	
	N	210	210
